# Caractéristiques sociodémographiques et sémiologiques de la sphère ORL des patients avec infection par le VIH/SIDA à Kinshasa, RD Congo

**Published:** 2010-11-24

**Authors:** Florent Nzuzi Kawashi, Benjamin Longo-Mbenza, Nzanza Richard Matanda, Augustin Nge-Okwe, Simon Mbungu Fuele

**Affiliations:** 1 Service d’ORL et de Chirurgie Cervico-Faciale, Cliniques Universitaires de Kinshasa, RD Congo; 2 Faculty of Health Sciences, Walter Sisulu University, Mthatha, South Africa; 3 Laboratoire d’Epidémiologie Clinique et Biostatistique, LOMO Medical, Kinshasa, RD Congo

**Keywords:** Afrique sub-Saharienne, céphalées, rhinorrhée, VIH/SIDA

## Abstract

**Background:**

L’absence de données congolaises relatives à l’épidémiologie, aux plaintes et signes cliniques de la sphère ORL dans l’infection par le VIH en consultation d’otorhinolaryngologie justifie l’initiation de la présente étude. L’objectif de notre étude était de décrire les aspects sociodémographiques et sémiologiques de la sphère ORL chez les patients avec infection à VIH/SIDA dans le service d’ORL de l’hôpital Général de Kinshasa, République Démocratique du Congo.

**Méthodes:**

Etude transversale réalisée entre Janvier et Avril 2009 dans le service d’ORL de l’Hôpital Général de Kinshasa, RDC.

**Résultats:**

Au total, 52 patients infectés par le VIH/SIDA et d’âge moyen de 40,6±13 ans ont été enquêtés. Le sexe féminin, la tranche d’âge de 30 -40 ans, les mariés et les veufs, les nantis et les plus scolarisés étaient les caractéristiques observées chez les patients infectés par le VIH/SIDA. Les céphalées et la rhinorrhée sous toutes ses formes, l’hypertrophie de la parotide, les adénopathies cervicales constituaient les aspects sémiologiques les plus rencontrés.

**Conclusion:**

La connaissance des aspects sociodémographiques et sémiologiques est indispensable dans le diagnostic précoce de l’infection par le VIH/SIDA.

## Introduction

Au début des années 1980, la pandémie du Syndrome d’Immunodéficience Acquise (SIDA), a été rapportée au monde sans connaissance de l’agent pathogène [1,2]. Cette forme tardive de la maladie est l’aboutissement de la progression de la déplétion immunitaire liée au virus de l’immunodéficience humaine (VIH). Cette pandémie frappe davantage l’Afrique subsaharienne et selon les données de l’ONUSIDA, 68% des 33,2 millions des personnes infectées par le VIH vivent dans cette région oésident moins de 10% de la population mondiale [3]. En République Démocratique du Congo (RDC), selon le Programme National de la Lutte contre le SIDA (PNLS), la séroprévalence dans la population générale est estimée à 4,04% [4].

Plusieurs facteurs sociodémographiques dont le sexe, l’âge, le niveau d’étude, la profession, la toxicomanie, le comportement à risque (tabagisme, excès d’alcool, partenaire multiple, non utilisation des préservatifs), l’homosexualité et les transfusions sanguines non testées sont incriminés dans l’acquisition de l’infection par le VIH /SIDA.

Dans les pays développés, le diagnostic de l’infection par le VIH/SIDA repose sur l’axe de laboratoire (classification CDC) [5] et l’axe clinique (Stades de l’OMS) [6]. Mais face au manque de ressources financières et des infrastructures de laboratoire dans les pays en voie de développement, l’OMS avait proposé les critères de Bangui basés sur la sémiologie et les affections opportunistes. Les seules manifestations de la sphère ORL et cervico-faciales reconnues comme forte suspectes d’infection par le VIH/SIDA sont les adénopathies cervicales, la candidose buccale et oro-pharyngée, la leucoplasie de la langue [7].

Les pathologies de la sphère ORL au cours de l’infection par le VIH/SIDA sont de plus en plus fréquentes et préoccupent plus les spécialistes en ORL, les médecins généralistes et les autres professionnels de santé en dehors des hôpitaux de référence. En effet, plus de la moitié des patients infectés par le VIH présentent des atteintes ORL et cervico-faciales [8].

Ces atteintes polymorphes peuvent se voir à tous les stades de l’infection par le VIH/SIDA; elles sont à l’origine d’un inconfort supplémentaire pour les patients et peuvent ainsi constituer le premier motif de consultation [7,8].

L’absence de données congolaises relatives à l’épidémiologie, aux plaintes et signes cliniques de la sphère ORL dans l’infection par le VIH en consultation ORL justifie l’initiation de la présente étude. L’intérêt de cette étude est l’utilisation ultérieure de tous les aspects épidémiologiques, des symptômes et des signes cliniques de la sphère ORL devant conduire à conseiller et effectuer le dépistage précoce de l’infection à VIH/SIDA. Ceci est d’autant plus justifié qu’en effet la République Démocratique du Congo ne possède que 20 spécialistes ORL pour une population générale de plus de 80 millions d’habitants.

L’objectif de la présente étude était de décrire les aspects sociodémographiques et sémiologiques en présence de l’infection par le VIH/SIDA chez un groupe de patients consultant dans le service d’ORL de l’hôpital Général de Kinshasa, République Démocratique du Congo.

## Methodes

Il s’est agi d’une étude transversale réalisée chez les patients examinés entre Janvier et Avril 2009. Le Service d’Oto-Rhino-Laryngologie (ORL) de l’Hôpital Général de Kinshasa, Commune Gombe, République Démocratique du Congo a servi de cadre à cette enquête. Et les autorités institutionnelles ont approuvé le protocole de la présente étude réalisées après consentement éclairé des adultes et des parents pour les enfants de moins de 18 ans, selon la Déclaration d’Helsinki II.

Etaient éligibles la série consécutive des patients examinés durant la période et le cadre de la présente étude. Ces patients ont tous accepté de participer à cette étude et d’effectuer une sérologie anti-VIH après counseling (taux de réponse de 100%).

Au cours de l’anamnèse, un questionnaire anonyme, structuré et standardisé a été présenté face à face pendant 30 minutes pour récolter les données épidémiologiques et les plaintes des patients. Les patients ont été ensuite examinés par le spécialiste en ORL pour décrire l’état des aires ganglionnaires, sous mandibulaires, trachéales et cervicales, état de la langue, des parotides et des amygdales. Aucun examen complémentaire n’était réalisé: le diagnostic n’étant pas parmi les objectifs de la présente étude. Tous les paramètres épidémiologiques et sémiologiques d’intérêt ont été colligés à l’aide des fiches précodées. Les paramètres épidémiologiques étaient les suivants: sexe, âge, migration interne ruro-urbaine, statut professionnel, commune de résidence, ethnie, niveau d’étude, état matrimonial et les sites géographiques d’origine. Les paramètres sémiologiques étaient les suivants: céphalées, otorrhée, éternuement, hypertrophie des amygdales, chatouillement oropharyngé, otalgie, état blanchâtre de la langue, hypertrophie des parotides et adénopathie cervico-faciale.

**Définitions opérationnelles**

L’ethnicité était définie par la langue et l’aire culturelle des patients: les Kongo (Province du Bas- Congo, et Bandundu), les Luba (Kasaïriental et Occidental), les Ngala (les Provinces de l’Equateur et le nord du Bandundu) et les Swahili (Provinces de Katanga, Nord Kivu, Maniema, Sud Kivu et la Province Orientale).

L’état de la langue était reconnu positif par la présence d’un enduit blanchâtre épais. Par contre l’état des amygdales et des parotides était défini par leur volume (hypertrophique contre normal).

Le niveau d’étude était divisé en bas (illettré, primaire) et en élevé (secondaire, supérieur, universitaire).

Le niveau socio-économique était élevé (indépendant, secteur privé et résidence urbaine) ou bas (sans revenu constant, résidence rurale).

**Analyses statistiques**

Les variables quantitatives ont été résumées sous forme de moyenne ±écart type, avec leurs extrêmes, mais les variables qualitatives sous forme de proportions (%).Les données ont été saisies sur microordinateur et analysées en utilisant le logiciel SPSS pour Windows version 13 (SPSS INC, Chicago, IL, USA).

## Results

Au total, 52 patients infectés par le VIH ont été enquêtés avec une prédominance féminine (42 femmes pour 10 hommes: sex ratio 4 femmes: 1 homme). Au total, 52 patients infectés par le VIH ont été enquêtés avec une prédominance féminine: 42 femmes pour 10 hommes.

**Figure 1:**
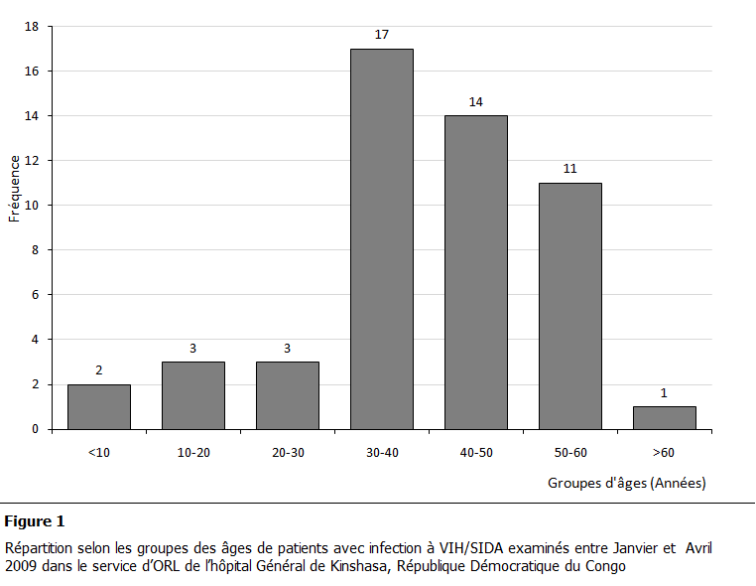
Répartition selon les groupes des âges de patients avec infection à VIH/SIDA examinés entre Janvier et Avril 2009 dans le service d’ORL de l’hôpital Général de Kinshasa, République Démocratique du Congo

L’âge moyen de ces patients était de 40,55±13 ans (extrêmes 8-65 ans). La tranche d’âge 30 -40 ans était la plus représentée dans la population étudiée (Figure 1). La fréquence de l’infection par le VIH était plus élevée chez les mariés 29% (n=14) et veufs 29% (n=14) suivis des célibataires 24,5% (n=12) et des divorcés 18% (n=9). La répartition de ces patients était égale parmi les groupes ethniques: 13 patients par groupe. Les patients infectés par le VIH étaient plus nombreux dans le niveau socio-économique élevé 71 % (n=37) que ceux de niveau bas 29% (n=15). Les patients avec niveau d’éducation élevé 77% (n=40) étaient plus fréquents que ceux ayant un niveau bas 23% (n=12). Le tableau 1 présente les symptômes ORL des patients examinés. La rhinorrhée sous toutes ces formes et les céphalées dominaient la symptomatologie de la sphère ORL suivies de l’obstruction nasale et des acouphènes. A l’inspection instrumentale, la rhinorrhée sous toutes ses formes était plus objectivée que la langue avec enduit blanchâtre, l’hypertrophie de la parotide et les adénopathies cervicales (Tableau 2). La répartition de la population d’étude selon le sexe, l’âge, l’état matrimonial, niveau d’étude et la profession était influencée (p<0,05) par la sérologie anti-VIH.

## Discussion

La présente étude a décrit les différentes caractéristiques sociodémographiques et sémiologiques chez les patients congolais avec infection à VIH. Le sexe féminin, la tranche d’âge entre 30-40 ans, les mariés, les veufs, le niveau socio-économique élevé et les ménagères étaient caractéristiques de la présence de l’infection par le VIH/SIDA dans la présente étude comme rapporté dans la littérature [6-21]. Les femmes africaines paupérisées sont souvent plus vulnérables que les hommes [12]. Le rapport ONUSIDA 2002 estime qu’en Afrique sub-saharienne deux fois plus de femmes sont infectées par le VIH/SIDA. Rares sont les études africaines qui rapportent une prédominance masculine dans l’infection à VIH/SIDA à cause de la transmission plus souvent hétérosexuelle [8]. Cette prédominance masculine est beaucoup plus nette dans les pays développés à cause de la transmission homosexuelle et la toxicomanie [9,14,15]. Les patients avec niveau socioéconomique élevé sont plus retrouvés dans notre étude ce qui n’a pas été le cas dans l’étude de Vignikin au Bénin qui constate que ce sont les couches socioprofessionnelles et à profession itinérante qui sont plus touchées. Au plan sémiologique, l’infection par le VIH a été caractérisée par des expressions polymorphes et variées [8,11]. Cette symptomatologie était dominée par la rhinorrhée sous toutes ses formes, l’obstruction nasale et les céphalées. Une association positive et significative a été démontrée entre la présence des céphalées, la rhinorrhée, l’hypertrophie de la parotide et les adénopathies cervicales et la présence de l’infection par le VIH/SIDA [12].

**Limites**

La présente étude a été limitée par sa na nature transversale. Ce qui ne permet pas de prouver une association causale comme le ferait les études longitudinales de cohorte.

## Conclusion

Les signes cliniques ORL sont suggestifs de la présence de l’infection à VIH sans distinction des stades cliniques de cette pandémie selon l’OMS. Ainsi la parfaite connaissance de ces aspects sociodémographiques et sémiologiques dans nos pays de l’Afrique Sub-saharienne est un atout majeur dans le dépistage précoce de l’infection par le VIH/SIDA.

## Conflit d’intérêts

Les auteurs ne déclarent aucun conflit d’intérêt.

## Authors’ contributions

Nzuzi Kawashi F: a conçu le thàme de l’étude et a récolté les cas, Longo-Mbenza B: a assuré la direction de la rédaction scientifique, Matanda Nzanza R: Membre de la rédaction scientifique, Nge Okwe et Mbungu Fuele: chargés la saisie et des analyses des données épidémiologiques.

## Figures and Tables

**Tableau 1:** Plaintes ORL chez un groupe de patients avec infection à VIH/SIDA examinés entre Janvier et Avril 2009 dans le service d’ORL de l’hôpital Général de Kinshasa, République Démocratique du Congo

**Variables d’intérêt**	**Nombre**	**%**
Céphalées	33	63,5
Chatouillement oro-pharyngé	6	11,5
Otalgies	14	26
Acouphànes	19	36,5
Otorrhée	15	28
Odynophagie	5	9,6
Obstruction nasale	34	65,4
Rhinorrhée sous toutes ses formes	40	76,9

ORL : Otorhinolaryngologie

**Tableau 2:** Données de l’inspection instrumentale ORL chez un groupe de patients avec infection à VIH/SIDA examinés entre Janvier et Avril 2009 dans le service d’ORL* de l’hôpital Général de Kinshasa, République Démocratique du Congo

**Variables d’intérêt**	**Nombre**	**%**
Rhinorrhée muco-purulente	9	22,6
Rhinorrhée purulente	21	52,5
Rhinorrhée postérieure	15	37,5
Langue avec enduit blanchâtre	15	28,8
Hypertrophie de parotide	6	11,5
Adénopathies cervicales	7	7,7

ORL : Otorhinolaryngologie
